# Anti-Cariogenic Potential of Lyophilized Cell-Free Supernatant of Probiotic *Pediococcus pentosaceus* GS4 against *Streptococcus mutans* and *Streptococcus anginosus*

**DOI:** 10.4014/jmb.2604.04005

**Published:** 2026-07-14

**Authors:** Sudaarsan Aruna Senthil Kumar, Asit Ranjan Ghosh

**Affiliations:** Microbial Molecular Biology Laboratory, Department of Integrative Biology, School of Bio Sciences and Technology (SBST), Vellore Institute of Technology (VIT), Vellore-632014, India

**Keywords:** Dental caries, Biofilm, Probiotics, LCFS, Virulence, Anti-cariogenic

## Abstract

*Streptococcus* species, particularly *Streptococcus mutans* and *Streptococcus anginosus*, are recognized globally for their role in causing dental caries and other dental issues. Current treatments using fluoride products and conventional antibiotics are often ineffective due to the resilience of biofilms on tooth surfaces. To overcome these challenges, novel approaches that eliminate these pathogens are much needed. In this regard, we have prepared CFS (cell-free supernatant), a postbiotic preparation of our probiotic strain *Pediococcus pentosaceus* GS4, and investigated the anticariogenic properties of its lyophilized form (LCFS). A quantity of LCFS (400 mg/mL) demonstrated bactericidal, antibiofilm, and anti-caries properties against *S. mutans* and *S. anginosus*. The physiological effectiveness of the LCFS was assessed in a simulated artificial salivary fluid (ASF), which showed biofilm inhibition of more than and around 65% for single-pathogen biofilms and above 60% for both-species biofilms, whereas the biofilm formation was inhibited by more than 65% and 60%, respectively, in the growth medium (BHIB). Again, mature biofilm disruption was higher in ASF than in the growth medium. These observations were confirmed by using light microscopy, scanning electron microscopy, and confocal microscopy. Additionally, LCFS reduced the cariogenic properties of these pathogens by lowering acid tolerance, reducing EPS production, and increasing sensitivity to H_2_O_2_. These findings suggest that such a simple, functional postbiotic preparation could be a promising anticariogenic agent for treating dental caries.

## Introduction

Dental caries, also known as tooth decay, is a common chronic disease that affects the majority of the world's population, regardless of their gender, age, or socioeconomic status [1]. According to the World Health Organization, dental caries affects an estimated 2.5 billion people worldwide and thus poses a significant public health concern [2]. It causes severe pain, discomfort, difficulty chewing, and tooth loss in the host [3]. Caries development is influenced by multiple factors, including cariogenic microflora, fermentable carbohydrates, and a susceptible host [4]. The oral environment serves as a bacterial niche, supporting a diverse and complex community that differs from that of the human gut [5].

Oral microflora accommodates various microbial genera of bacteria, fungi, viruses, archaea, and protozoa [6]. Bacterial genera that comprise the oral microbiome include the Streptococci group, which is predominantly found in the oral cavity, particularly on dental plaque [7]. Among Streptococci, *Streptococcus mutans*, a Gram-positive bacterium, is considered the major causative agent for dental caries [8]. *S. mutans* possesses several virulence factors, like adherence to tooth surfaces, acid tolerance, acid production, synthesis of extracellular polysaccharides (EPS), and biofilm formation [9]. The pathogenicity of *S. mutans* is linked to active carbohydrate metabolism, resulting in lactic acid production and a pH drop in plaques in the oral environment, leading to enamel erosion [7]. These characteristics allow *S. mutans* to colonize in the oral environment and cause effective infections.

Following primary colonization and biofilm formation, commensal biofilms are replaced by pathogenic biofilms, leading to disease progression [10]. Again, *S. mutans* biofilms favour other pathogenic bacteria by creating a microenvironment and providing nutrients released during metabolism [7]. Likewise, *Streptococcus anginosus*, a member of the “Milleri group” and an opportunistic pathogen, has emerged as a potent microbe capable of inducing abscess formation and deep carious lesions [11]. Both *S. mutans* and *S. anginosus* survive well in highly acidic conditions (pH 3.5), form biofilms, and cause dental caries, which is followed by periodontitis [12-14].

Current treatment strategies include re-mineralization in the initial stages of infection with fluorides, followed by restoration with dental fillers; crowns for moderate infection; and endodontic therapy for severe decay to prevent disease progression [15]. Antibiotics have not been found to be much effective in the treatment of oral caries [16]. At present, commonly used anti-caries agents are fluoride products, xylitol, triclosan, and Chlorhexidine. Although fluoride exhibits anticaries effects, increasing fluoride levels alters the oral microflora and promotes overall resistance [17, 18]. Therefore, there is a need to explore new approaches for better oral health management.

In recent years, postbiotics have attracted considerable attention among researchers for their widespread applications in conferring health benefits to the host. Probiotics are live microorganisms, whereas postbiotics are the preparation of inanimate microorganisms and their components that favor the well-being of the host [19]. Postbiotics have gained significant recognition as an alternative to probiotics, offering additional advantages, including stability, longer shelf life, and ease of delivery [20]. Studies showed that cell free supernatant (CFS) produced by *Lactobacillus* species possesses bactericidal and anti-adherence effects against the cariogenic bacteria [21, 22].

In this study, we employed a postbiotic preparation derived from the probiotic strain *Pediococcus pentosaceus* GS4 (MTCC 12683), isolated from the Indian fermented food Khadi and characterized in our laboratory. The strain was well studied for its probiotic properties, anti-cytotoxic, and anti-xenotoxic effects and considered to be safe. This preparation exhibits anti-adherence and anti-biofilm activities and disrupts mature biofilms formed by *Streptococcal* species. Additionally, the assays were conducted in artificial saliva at two pH levels, representing a healthy oral environment and a caries-progressive environment, and were analyzed using various microscopy. The results demonstrate antibiofilm and biofilm-disruptive effects, suggesting that the lyophilized cell-free supernatant (LCFS) of our probiotic strain GS4 could be a promising candidate to address the problem.

## Materials and Methods

### Chemicals and Reagents

All the chemicals and reagents employed in the study were procured from HiMedia (India) and Sisco Research Laboratories (SRL), (India). Culture plates used for the *in vitro* assays were obtained from Tarsons, India.

### Microbial Strains and Culture Conditions

The reference strains used in this research were oral pathogens, *Streptococcus mutans* (ATCC 25175) and *Streptococcus anginosus* (MTCC 1929), obtained from ATCC and MTCC, respectively, and a probiotic strain, *Pediococcus pentosaceus* GS4 (MTCC12683) [23] (Ghosh and Ghosh, 2025), procured from our laboratory. Pathogenic strains were cultured in Brain Heart Infusion broth (BHIB-pH 7.4). *S. mutans* and *S. anginosus* were grown in BHIB for 14 h at 37ºC with a cell density of 10^8^ CFU/mL, equivalent to 0.4 at 600 nm, and were used throughout the study.

### Preparation of Cell-Free Supernatant and Lyophilization

Probiotic *P. pentosaceus* GS4 was cultured in De Man, Rogosa, and Sharpe (MRS) broth and incubated at 37ºC for 48 h. Following incubation, the CFS was obtained by centrifugation at 8000 rpm for 10 min at 4°C, then filter-sterilized through a 0.22 μm sterile filter. The filtered CFS (pH 4.2) was lyophilized. The LCFS was like flakes, dissolved in sterile 1× PBS (Phosphate-Buffered Saline) and used in subsequent assays.

### Determination of Antimicrobial Activity of the LCFS against Oral Pathogens

The antimicrobial activity of LCFS against oral pathogens was determined using the pour plate method [24]. Briefly, *S. mutans* and *S. anginosus* were sub-cultured in BHIB. Following their growth, a 1 mL aliquot of the culture was seeded into 24 mL of BHI agar, which was then poured onto a plate. After solidification, wells were punctured using a sterile disposable tip. An aliquot of 100 μL of Erythromycin-50 μg/mL (E-50 as positive control), vehicle control (1× PBS), and LCFS (100 to 500 mg/mL) were added to their respective wells. The plates were incubated at 37°C for 24 h, and the zone of inhibition (ZOI) was subsequently measured. Here, erythromycin was used as a positive control in our study due to its broad-spectrum activity against gram-positive pathogens, which allows for a standardized comparison between the LCFS.

### pH Neutralization, Heat Stability, and Proteinase K Treatment of LCFS

The pH of LCFS was adjusted to 7.0 using 1N NaOH. It was then evaluated for its antimicrobial property by the agar well diffusion method and metabolic activity by MTT assay. Similarly, the LCFS was subjected to proteolytic enzymatic digestion using proteinase K (1 mg/mL) and incubated at 37°C for 2 h, followed by heating at 100°C to inactivate the enzyme. For the thermal stability determination, the LCFS was treated at 60°C, 80°C, 100°C, and 121°C, respectively.

### Determination of the Viability of *S. mutans* and *S. anginosus*

The minimum inhibitory concentration (MIC) of LCFS was determined using the broth microdilution technique in a 24-well microtiter plate. Briefly, 1% of the overnight cultures of both test pathogens were sub-cultured into 1 mL of BHIB each in a 24-well microtiter plate, with and without LCFS, and incubated at 37°C overnight. Erythromycin (50 μg/mL) was used as a positive control. Following overnight incubation, 100 μL of MTT (0.5 mg/mL) was added, and incubated at 37°C in the dark for 4 h. Subsequently, the optical density of the suspension was measured at 570 nm using a microplate reader, and metabolic activity was determined [25].

### Kill Curve Analysis of LCFS against *Streptococcus* spp.

The growth-inhibitory effect of the LCFS was assessed using kill curve analysis. Time-kill kinetics were determined by exposing *S. mutans* and *S. anginosus* individually to the LCFS for 24 h. In the assay, *S. mutans* and *S. anginosus* at a cell density of 10^8^ were added to BHIB with or without LCFS (400 mg/mL, pH 3.8), and broth without LCFS served as a control. Every 2 h, the samples were spotted onto BHI agar plates to determine the reduction in CFU over time, and the plates were incubated at 37°C for 24 h. Colonies were enumerated, and a graph was plotted with Log CFU versus time[26, 27].

### Assessment of LCFS in Streptococcal Biofilm Inhibition

The efficacy of LCFS in preventing biofilm formation of *S. mutans*, *S. anginosus*, and dual species (*S. mutans* + *S. anginosus*) was evaluated through a biofilm inhibition assay following [28]. Briefly, to a sterile 96-well plate, 100 μL of BHI broth, 50 μL of cultured cells (in case of dual species biofilm, an equal amount of culture was suspended in a 1:1 ratio), and 50 μL of the LCFS were added to respective wells and incubated at 37°C for 24 h. Wells without LCFS served as the control, and wells with E-50 served as the positive control. Following incubation, the media was removed, the cells were washed twice with 1× PBS to remove non-adhered cells, and then 200 μL of 0.4% crystal violet was added. After 30 min, crystal violet was removed, and the wells were washed twice to remove excess stain. Following removal of excess stain, 70% alcohol was added to the corresponding wells, and OD was recorded at 595 nm [28]. The formula used to determine biofilm inhibition:

$$\text{Percentage of biofilm inhibition:}\frac{\text{Control OD}-\text{Test OD}}{\text{Control OD}}\times 100$$

Control OD- OD of the biofilm formed without any treatment

Test OD – OD of the biofilm formed with treatment (E-50, LCFS)

### Biofilm Disruption Potential of LCFS Using Crystal Violet Staining

The ability of LCFS in the disruption of pre-formed Streptococcal biofilms was assessed by culturing *S. mutans*, *S. anginosus*, and dual species in a sterile 24-well plate with 1 mL of BHI broth for 24 h at 37°C. After 24 h of incubation, the spent media was removed, and the culture was supplemented with fresh media containing LCFS, then incubated for 24 h. Wells without LCFS served as the negative control, and wells with E-50 served as the positive control. Following the incubation, the media was removed, and the wells were washed twice to remove unbound cells. Further, 0.4% crystal violet was added to the biofilms formed in the respective wells. After 30 min, the stain was removed, the sample was washed twice with 1× PBS, then 70% alcohol was added, and the sample was read at 595 nm [29]. Biofilm disruption was calculated using the formula:

$$\text{Percentage of biofilm disruption}:\frac{\text{Preformed biofilm OD-Treated biofilm OD}}{\text{Preformed biofilm OD}}\times 100$$

### Visualisation of Mature Biofilm Disruption by Light Microscopy

To visualize biofilms formed by pathogens in mono- and dual-species conditions, sterile 1×1 cm glass slides were placed in a 24-well plate and incubated for 24 h at 37°C. Following incubation, the spent media and non-bound cells were removed, and fresh media were seeded on the developed biofilm over the glass slides. LCFS was added to the respective wells, and the wells were incubated for 24 h at 37°C. After incubation, the media was removed, and unadhered cells were washed with 1× PBS, then 0.4% crystal violet was added and incubated for 30 min. Further, the slides were rinsed twice with 1× PBS and visualized under a light microscope at 100× magnification [28].

### Preparation of Artificial Salivary Fluid (ASF)

To evaluate the potential of LCFS in preventing and disrupting Streptococcal pathogenic biofilms, artificial saliva was prepared using the chemical components that mimic the oral environment (Table 1) [30]. The elements were dissolved in sterile H_2_O, the pH was adjusted, and the solution was sterilised using a 0.22 μm filter. To evaluate the ability of LCFS in the caries condition and normal oral environment, the Artificial saliva was prepared at two pH levels: 5.5, which mimics active dental caries, and 6.8, which mimics the resting mouth condition [26]. During the study, ASF was maintained at room temperature (25±1°C RT).

### Evaluating the Biofilm Inhibition and Disruption Ability of LCFS in ASF

In addition to the BHIB condition, biofilm inhibition and disruption studies were performed in ASF at pH 5.5 and 6.8 conditions. Biofilm inhibition was performed similarly to the above-mentioned biofilm inhibition assay, and biofilm disruption with the disruption assay performed earlier with BHIB. Here, the pathogens mono as well as dual species were inoculated in the prepared ASF (5.5 and 6.8) separately, to evaluate the ability of LCFS in preventing biofilm formation as well as disrupting the preformed biofilm.

### Enumeration of Viable Count from Treated Biofilm

The effect of LCFS on viable biofilm cells was determined using the colony-count spotting method (Miles and Misra technique) across all three growth conditions.

Biofilms were developed in a 24-well plate for 24 h, as described above in the biofilm disruption section. Here, pre-formed biofilms were treated with LCFS for 24 h. Following that, viable cells were harvested, suspended in 1× PBS, serially diluted, spotted onto BHI agar plates, and incubated at 37°C for 24 h [31, 32]. Later, the number of viable cells was calculated, and a Log10 CFU/mL graph was plotted.

### Assessing the Effect of LCFS on Adherence

An adherence assay was conducted to assess LCFS's ability to inhibit *Streptococcus* spp. adherence. Briefly, both test strains were cultured in BHIB without (control) and with LCFS (treated), in ASF 5.5 and ASF 6.8 in glass tubes, and incubated by tilting them to 30º angle at 37°C for 24 h. Following incubation, the free-floating media and cells were removed without disturbance, and the adherent cells were collected by rinsing them with sterile 1× PBS. OD was measured at 600 nm for both control and treated cells [8, 33]. Here, E-50 was used as the positive control, and the group without any treatment served as the negative control. The percentage of adhesion was calculated using the formulae:

$$\text{Percentage of adhesion =}\frac{\text{OD of control-OD of treated}}{\text{OD of total control}}\times 100$$

Control OD- OD of the adhered cells to the glass tube without treatment

Test OD – OD of the adhered cells with treatment (E-50, LCFS)

### Impact of LCFS on Cell Surface Hydrophobicity

To investigate the effect of LCFS on cell-surface hydrophobicity, test pathogens were cultured under all three conditions, with and without LCFS, for 24 h at 37°C. After incubation, cells were collected by centrifugation, washed twice with 1× PBS, and resuspended in 1× PBS. Later, the suspension was adjusted to an OD of 1, mixed with toluene at a 1:3 (toluene: cell suspension) ratio, and vortexed for 5 min. After vortexing, the mixture was allowed to stand at room temperature for 10 min to facilitate separation between the toluene and aqueous phase. After phase separation, the OD of the aqueous phase was recorded at 600 nm [34]. Relative hydrophobicity was determined using the formula:

$$\text{Percentage of hydrophobicity}=\left(1-\frac{\text{OD at 600 nm after vortexing}}{\text{OD at 600 nm before vortexing}}\right)\times 100$$

### Quantification of Exopolysaccharide from Biofilms

EPS from Streptococcal biofilms in mono-and dual-state cultures was quantified using the phenol-sulfuric acid method to determine total carbohydrate content. Briefly, biofilms were grown in 6-well plates with or without LCFS for 24 h. Following incubation, the biofilms were collected with 1× PBS and treated with 5% phenol. Subsequently, 0.2% hydrazine sulfate dissolved in concentrated sulfuric acid was added to the above suspension, which was then incubated for 1 h in the dark. Later, the suspension was centrifuged at 10000 rpm for 10 min, and the resulting supernatant was quantified spectrophotometrically at 490 nm [29].

### Evaluating the Role of LCFS on Acid Tolerance

The acid tolerance of both *S. anginosus* and *S. mutans* was assessed by measuring viability after exposure to pH 5. Bacterial cells were collected at their mid-logarithmic phase by centrifugation at 8000 rpm for 10 min. Following centrifugation, the cell pellets were suspended in BHI medium at pH 5.0 with or without LCFS for 2 h at 37ºC. Following incubation, the cells were spotted onto BHI agar plates and incubated at 37ºC for 24 h to determine the viable count [35].

### Effect of LCFS on H_2_O_2_ Sensitivity of *Streptococcal* spp.

A hydrogen peroxide sensitivity assay was performed to assess the survival ability of the *S. mutans* and *S. anginosus*. For that, test strains were grown in BHIB, in the presence as well as absence of LCFS, and then the cells were collected by centrifugation at 8000 rpm for 10 min at 4°C. Later, the cells were resuspended in sterile 1× PBS and then spread onto BHI agar plates. Later, wells were punched, and 25 mM and 50 mM of H_2_O_2_ were added to the wells, and the plates were incubated for 24 h at 37°C [36].

### LIVE/DEAD Analysis of Biofilm by Confocal Laser Scanning Microscopy

Biofilms of dual species were grown over glass slides for 24 h at 37°C in the 24-well microtiter plate. Later, the spent media was removed and seeded with fresh media. LCFS and E-50 were added to their respective wells and further incubated for 24 h at 37°C. Following incubation, the spent media were removed, and the slides were washed three times with sterile 1× PBS to remove unbound cells. After washing, they were stained with SYTO 9 and propidium iodide (PI) and incubated in the dark for 15−20 min. Subsequently, the excess dye was removed by washing with 1× PBS. The slides were air-dried and visualized under a Confocal Laser Scanning Microscope (Fluoview Fv3000, Olympus, Japan) [37].

### Scanning Electron Microscopic Studies

Biofilm was visualized using a scanning electron microscope by developing dual-species biofilms of both pathogens on a 1 × 1 cm glass slide. Biofilm development was performed as described earlier in the disruption assays for light microscopy. After biofilm development, the spent media were removed, and the slides were fixed in 2.5% glutaraldehyde for 1 h. Subsequently, the samples were dehydrated in ethanol at 50%-100% ethanol, followed by gold sputtering, and the slides were visualized under SEM [32].

### Statistical Analysis

All the experiments were carried out in triplicate and reported as mean ± standard deviation. GraphPad Prism was used to plot the graphs in the study, and statistical significance was assessed using analysis of variance (ANOVA) followed by Dunnett’s post-hoc test. The *p*-values < 0.05 were considered statistically significant.

## Results

### Anti-Bacterial Property of LCFS against *Streptococcal* spp.

**Fig. 1:**
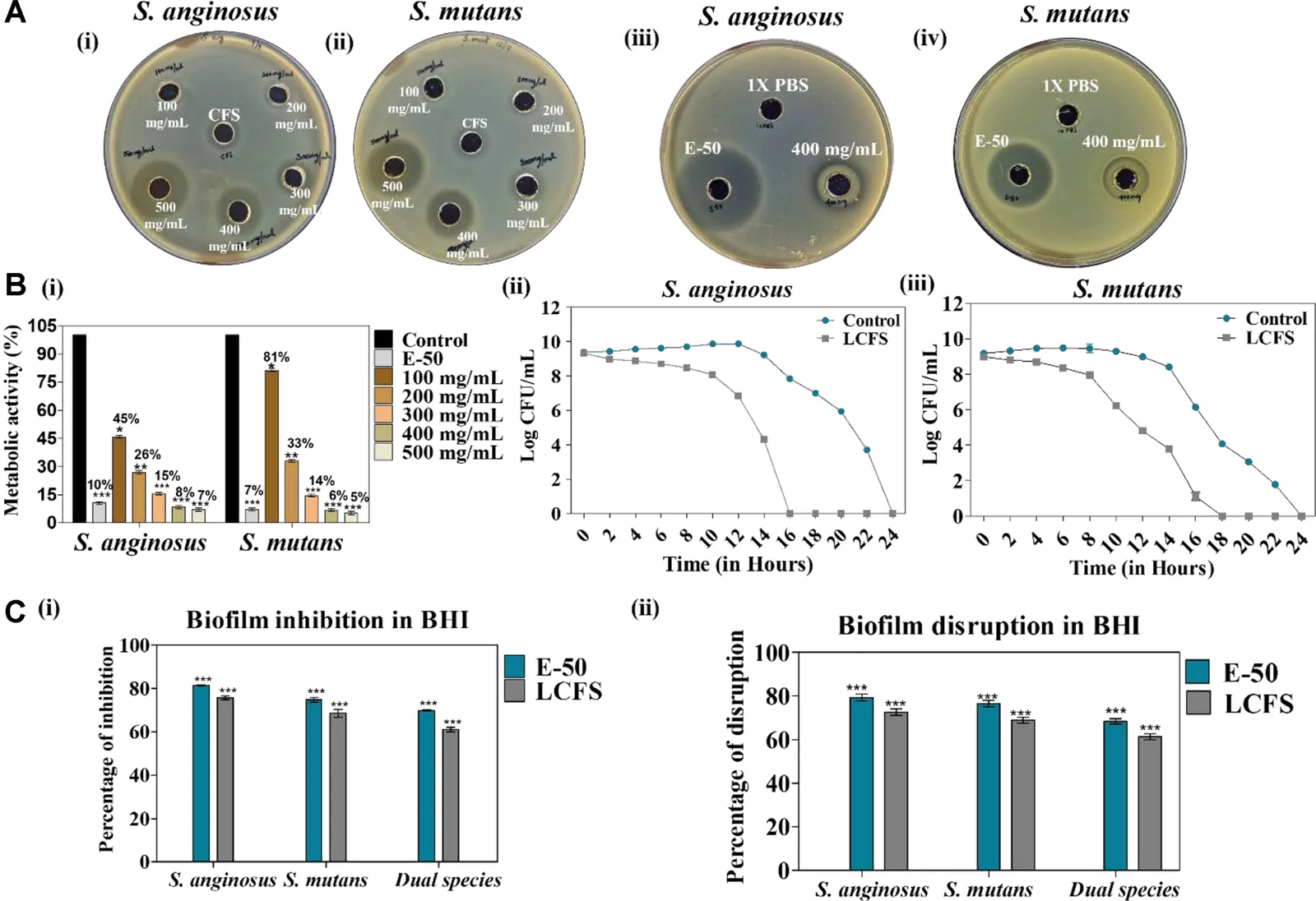
(**A**) (**i**) Antibacterial activity of LCFS against *S. anginosus*, (**ii**) *S. mutans*, and (**iii, iv**) comparison of LCFS with positive control and negative control employed in this study against the test pathogens. (**B**) (**i**) Assessment of metabolic activity of *S. anginosus* and *S. mutans* in the presence as well as absence of LCFS with varying concentrations. The data is presented in graphical format with the error bar representing standard deviation and * representing the *p*-value <0.01. (**iii**) The kill-curve analysis of 400 mg/mL LCFS on the growth of *Streptococcal* sp. (**C**) (**i**) The biofilm inhibition of LCFS (400 mg/mL) in BHIB medium for individual and dual species biofilm by crystal violet assay, with E-50 used as a positive control, and (**ii**) pre-formed biofilm disruption of LCFS.

The LCFS of probiotic GS4 was assessed for antibacterial activity using the pour plate method. LCFS with increasing concentration (100−500 mg/mL) showed ZOI between 11 and 21 mm against both pathogens (Table 2). The ZOI for *S. anginosus* at a 400 mg/mL concentration was 15 ± 0.20 mm (Fig. 1A-i), and for *S. mutans*, it was 16 ± 0.25 mm (Fig. 1A-ii). Erythromycin 50 μg/mL was used as a positive control, which showed a 30 ± 0.36 mm clear zone for *S. anginosus* and 23 ± 0.20 mm for *S. mutans*, as shown in Fig. 1A-iii and iv.[Table T3]

### Effect of LCFS on the Metabolic Activity of *S. anginosus* and *S. mutans*

LCFS was tested for its effect on the metabolic activity of oral pathogens using the MTT cell viability assay. The results showed that LCFS with 100 to 300 mg/mL concentration had no effect on viability, which resulted in the formation of formazan crystals. At LCFS concentrations of 400 mg/mL and higher, pathogen viability was inhibited, which was evident by the absence of formazan formation after addition of MTT and also with no viability (Fig. 1B-i). Based on these results of the agar well diffusion and MTT cell viability assays, 400 mg/mL was used as the working concentration in subsequent assays.

### Effect of pH, Thermal Stability, and Proteinase K on LCFS Anti-Bacterial Activity

Treated LCFS showed reduced antibacterial property and increased viability (% metabolic activity). This indicates that the pH, temperature, and proteinase-K treatment denatured some metabolites present in LCFS (Fig. 1B-i).

### Effect of LCFS on *Streptococcal* spp. Growth – Bactericidal Activity

LCFS was assessed for its bactericidal effect against the planktonic cells of each pathogen individually. Proliferation of *S. mutans* and *S. anginosus* was evaluated over 24 h by determining the CFU log reduction method for every 2 h starting from 0 to 24 in the presence as well as absence of LCFS. Results showed complete inhibition (no growth) after 16 h of exposure to LCFS in *S. anginosus* and after 18 h in *S. mutans* (Fig. 1B-ii). It was evident that a 400 mg/mL concentration inhibited the growth of both pathogens.

### Anti-Biofilm Property of LCFS

Streptococcal biofilms are well known for their ability to cause dental caries and a wide range of infections. As we observed, LCFS (400 mg/mL) inhibited the growth of oral Streptococcal pathogens, so its biofilm-inhibition potential against *S. anginosus* and *S. mutans*, and in dual-species (*S. anginosus* + *S. mutans*) biofilms, was examined. It is observed that in the presence of LCFS, the biofilm formation was inhibited by 75.7 ± 0.89% for *S. anginosus*, 68.5 ± 1.75% for *S. mutans*, and 61.1 ± 1.07 % for mixed biofilms in comparison to the use of E-50 (81.4 ± 0.24 %, 74.8 ± 1.13%, and 69 ± 0.31%), respectively (*p* < 0.05). Thus, the results indicate that LCFS is effective at preventing biofilm formation (Fig. 1C-i).

### Biofilm Disruption Ability of LCFS

In addition to inhibition, the LCFS was evaluated for its ability to disrupt mature biofilms of the oral pathogens used in this study. Notably, the LCFS demonstrated strong disruption potential against *S. anginosus* biofilm (72 ±1.47 %), followed by *S. mutans* (68 ± 1.37 %) and dual-species biofilm (61 ± 1.32%), compared with E-50 (79 ± 1.64%, 76.4 ± 1.49%, 68 ± 1.22%) (*p* < 0.05) (Fig. 1C-ii).

### Effect of LCFS on Biofilm Inhibition and Disruption in Artificial Salivary Fluid (ASF)

**Fig. 2:**
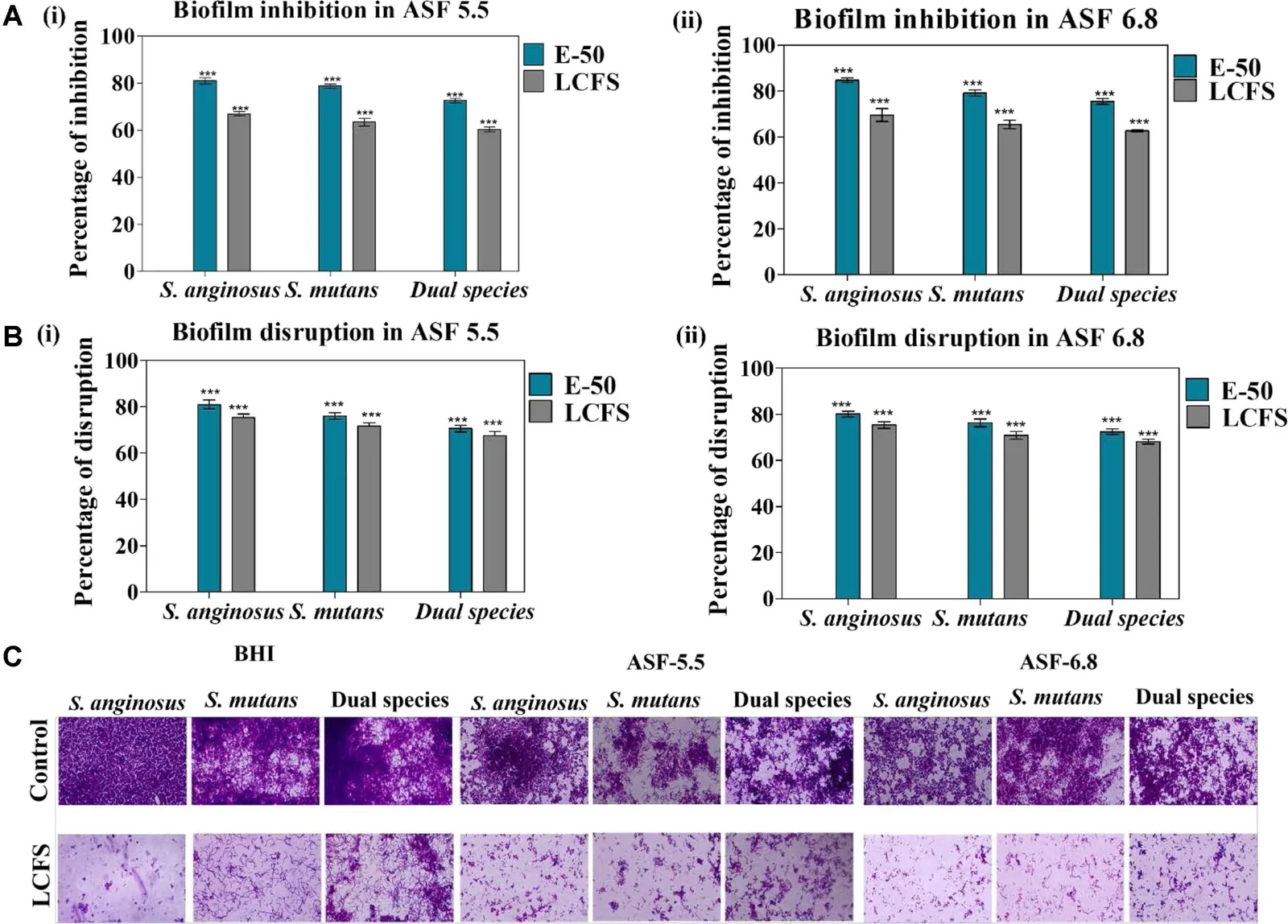
(**A**) (**i, ii**) The biofilm inhibition ability of LCFS against the individual and mixed species biofilm in simulated conditions in both ASF 5.5 and ASF 6.8 by crystal violet assay. (**B**) (**i, ii**) The ability of LCFS in the disruption of preformed biofilm in ASF 5.5 and ASF 6.8 by CV assay. (**C**) Light microscopic studies on the disruption efficacy of LCFS in all three conditions for both individual and mixed species biofilms, visualized at 100X magnification

Considering the human oral environment, ASF (pH 5.5 and 6.8) was formulated [30], and the LCFS was evaluated for its antibiofilm potential. Inhibition of biofilm formation using E-50 in ASF-5.5 exhibited nearly 80% biofilm inhibition in both individual- and dual-species biofilms. In contrast, the LCFS-treated biofilm inhibition rate was 67.04 ± 0.90 % for *S. anginosus*, 63.41 ±1.70% for *S. mutans*, and 60.39 ± 0.99 % in dual species biofilm, as shown in (Fig. 2A-i). Similarly, at the ASF-6.8 pH condition, E-50 showed 75% biofilm inhibition across all three biofilms. LCFS treatment has shown >65% biofilm inhibition for single-pathogen biofilms and 62% for dual-species biofilms (*p* < 0.05) (Fig. 2A-ii). Apart from inhibition, the mature biofilm disruption study showed varying levels of disruption. LCFS treatment disrupted 75.27 ± 1.53% of the biofilm formed by *S. anginosus*, 71.51 ± 1.42% by *S. mutans*, and 67.34 ± 1.92 % by dual species, while E-50 treatment exhibited an average of 75% disruption across all conditions (*p* < 0.05) (Fig. 2B). The results suggest the significant anti-biofilm potential of LCFS in both ASF conditions.

### Microscopic Analysis

LCFS-treated mature biofilms in all three conditions (BHIB, ASF-5.5, and ASF-6.8) were examined under a light microscope. The control samples showed dense, well-structured biofilms resembling a complete matrix, whereas the LCFS-treated slides showed less-dense biofilms, indicating the potential to disrupt preformed, mature biofilms (Fig. 2C).

**Fig. 3:**
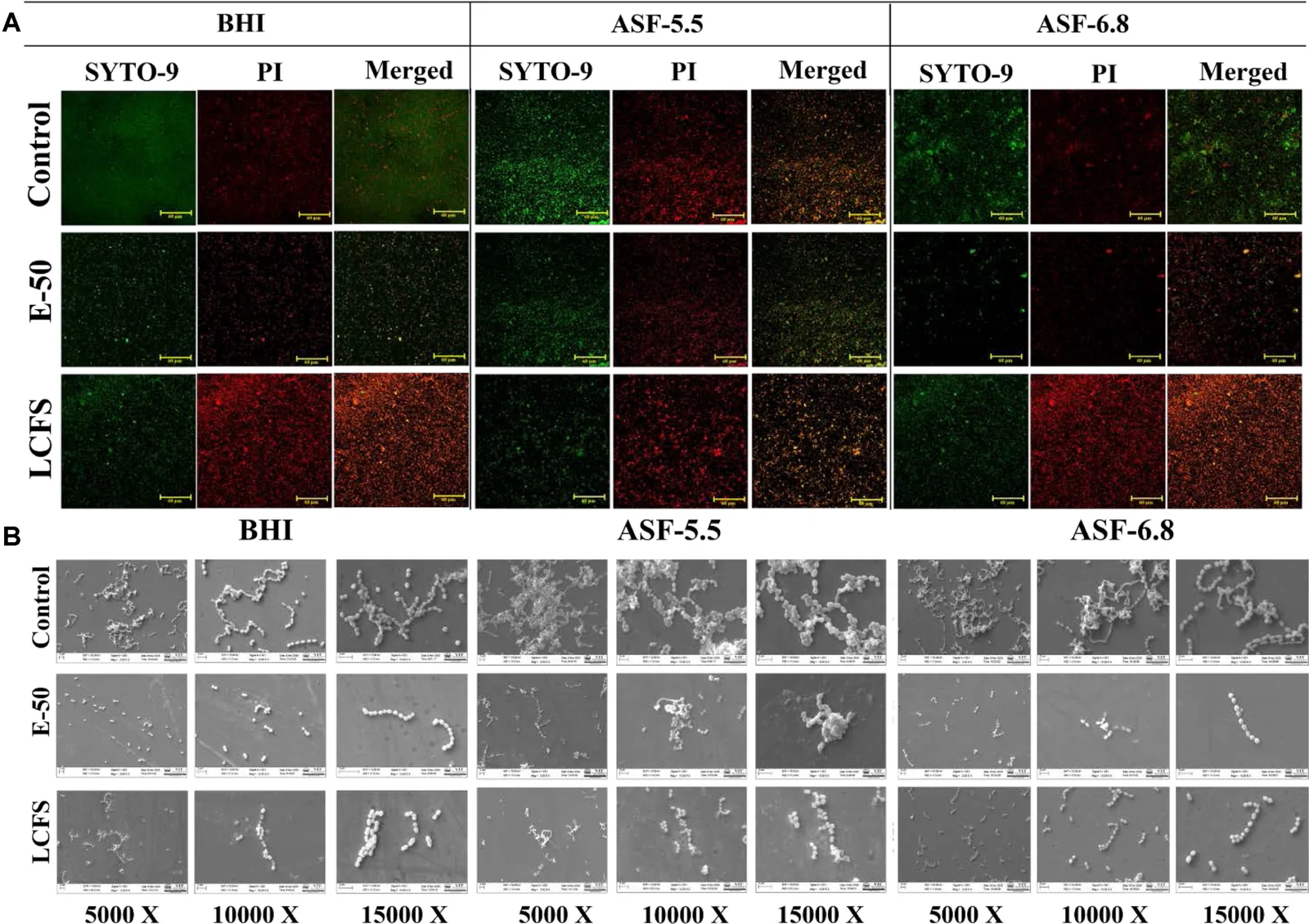
(**A**) CLSM micrographs representing the live and dead cells residing in the mixed species mature biofilm with and without LCFS in all three conditions. (**B**) Disruption potential of LCFS over the preformed mixed species biofilms visualized through scanning electron microscopy with 5000X, 10000X, and 15000X magnification.

In confocal laser scanning electron microscopy, live bacterial cells were observed in greater numbers than dead cells in control biofilms. But when treated with LCFS, it was evident that, across all three growth conditions in the dual-species biofilm, the LCFS treatment had a higher proportion of dead cells than live cells, as shown in Fig. 3A.

Similarly, SEM analysis was performed to assess the disrupting potential of LCFS and to examine detailed structural changes in dual-species biofilms of *S. anginosus* and *S. mutans*. Here, a dual-species biofilm was developed on the glass surface under all three conditions (BHIB, ASF 5.5 and ASF 6.8), mimicking the oral environment, assuming that the normal flora and single or multiple pathogens form biofilms under cariogenic conditions. Results from SEM analysis revealed numerous clusters of *Streptococcus* species across all three growth conditions without LCFS treatment. Clusters were observed more frequently at ASF-5.5; this may be due to optimal pH conditions during caries infection. In LCFS-treated samples, single chains of *Streptococcus* and some microcolonies were observed in all three conditions, thus visually confirming LCFS’s ability to disrupt mature biofilm.

### Effect of LCFS on Adherence Property of *S. mutans* and *S. anginosus*

**Fig. 4:**
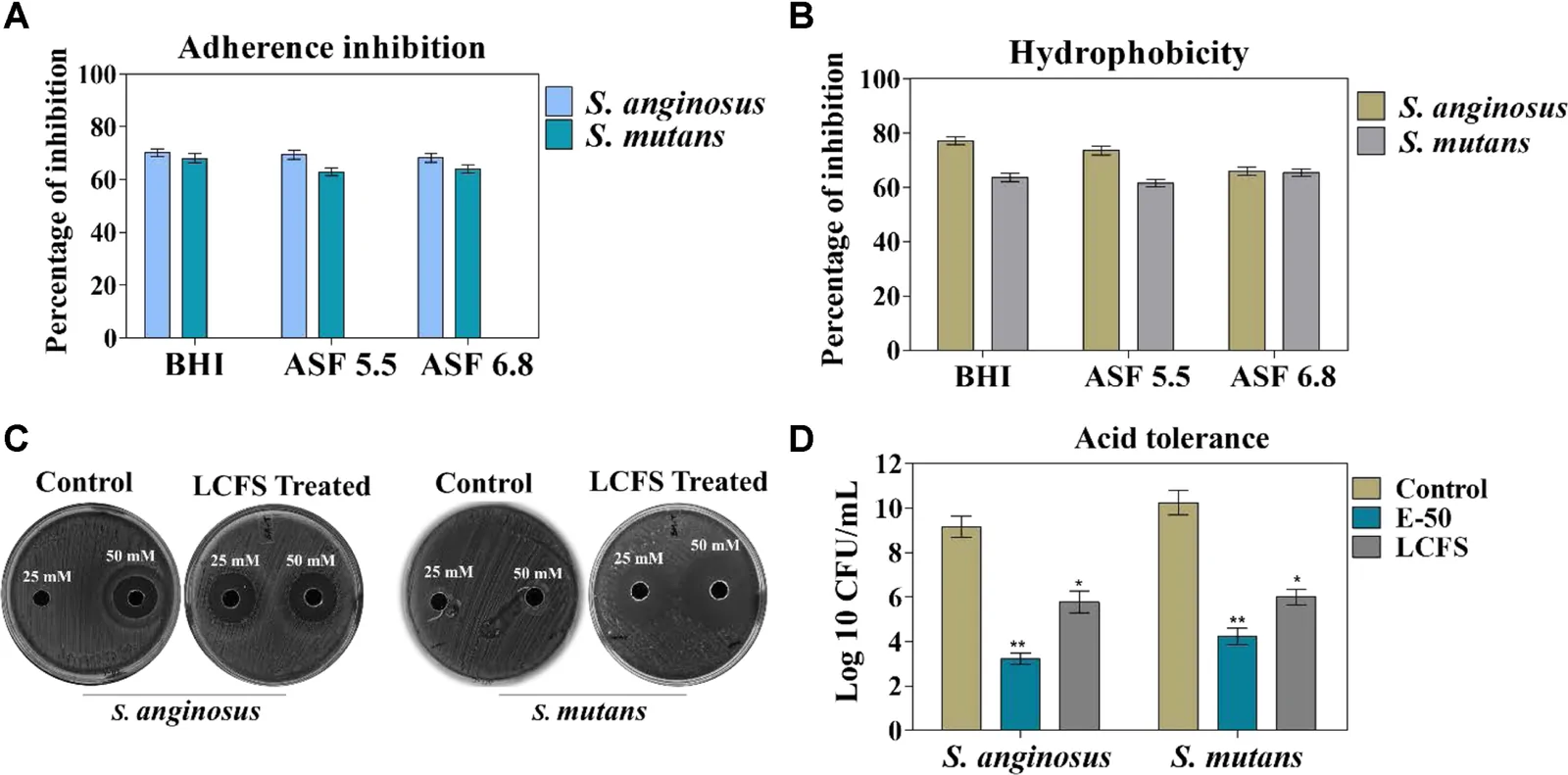
(**A**) The impact of LCFS on *S. anginosus* and *S. mutans* adherence to the hard surfaces is depicted in bar graphs representing the inhibition percentage. * represents the *p*-value <0.05. (**B**) The influence of LCFS on cell surface hydrophobicity of *Streptococcal* spp. represented in bar graph with percentage inhibition. (**C**) The plate images depicting the role of LCFS on sensitizing the *S. anginosus* and *S. mutans* towards hydrogen peroxide H_2_O_2_ (25, 50 mM). (**D**) The impact of LCFS on *Streptococcus* sp. acid tolerance ability. Compared with the untreated group, LCFS-exposed cells had fewer CFUs, as shown in the Log reduction graph.

Adherence of pathogenic bacteria to tooth surfaces is the initial step in biofilm formation. Here, the anti-adherence efficacy of the LCFS was assessed against *S. mutans* and *S. anginosus* in artificial salivary conditions and in growth media (BHIB). The results show that LCFS reduced adherence compared with untreated cells. Adherence inhibition was found to be above 60% in both *S. mutans* and *S. anginosus* in growth media and in ASF, indicating that LCFS would substantially reduce the pathogenic bacteria adherence to surfaces (Fig. 4A).

### Effect of LCFS on *Streptococcal* spp. Hydrophobicity

Hydrophobicity is a key factor that enables bacteria to adhere to the tooth surface and initiate biofilm formation. In general, *Streptococcal* spp. are highly hydrophobic. Hydrophobicity was calculated by measuring the number of cells adhered to toluene. Here, the assay results indicated that untreated cells had a higher affinity for toluene. In contrast, the affinity was reduced by more than 65% in LCFS-exposed cells for *S. anginosus* across all three growth conditions (BHIB, ASF 5.5, ASF 6.8) and by more than 60% for *S. mutans* as shown in Fig. 4B. These results indicate that LCFS significantly reduces the hydrophobicity of the pathogens, compromising their ability to form biofilms.

### Effect of LCFS on H_2_O_2_ Sensitivity

In *S. anginosus*, control cells exhibited resistance to 25 mM H_2_O_2_ and were sensitive to 50 mM with 17 ± 0.2 mm of clear zone, whereas the LCFS-treated cells were sensitive to both concentrations of H_2_O_2_ with 20 ± 0.14 mm for 25 mM and 23 ± 0.07 mm of clear zone for 50 mM concentration. Similarly, in *S. mutans*, control cells were resistant to both H_2_O_2_ concentrations. However, treated cells were sensitive to both, with 20 ± 0.14 mm for 25 mM and 32 ± 0.21 mm for 50 mM H_2_O_2_ (Fig. 4C). These results clearly demonstrate that LCFS treatment has sensitized these pathogens to H_2_O_2_.

### Effect of LCFS on Acid Tolerance

*Streptococcus* species, particularly *S. anginosus* and *S. mutans*, are tolerant to acidic conditions. Here, the acid tolerance of both species was determined individually at pH 5. In the control, bacterial cells were observed even at higher dilutions, whereas in LCFS-treated cells, up to a 4-log reduction in *S. anginosus* and a 3-log reduction in *S. mutans* were observed. Similarly, E-50-treated cells showed a 7-log reduction in *S. anginosus* and a 5-log reduction in *S. mutans* (Fig. 4D). The results showed that LCFS can inhibit the acid-tolerance trait of these two pathogens, which is primarily responsible for tooth decay.

### LCFS Reduces the Viable Cell Count in Biofilm

**Fig. 5:**
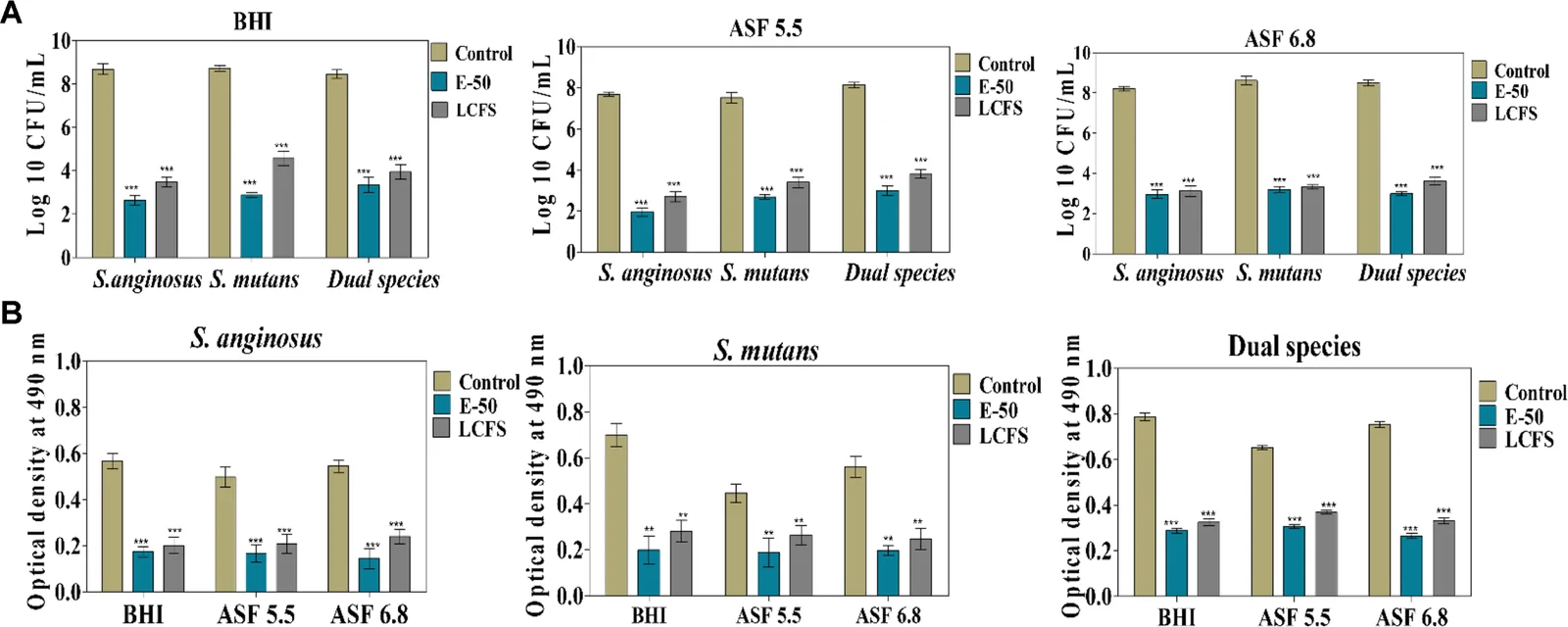
(**A**) The influence of LCFS on the viability of cells residing in the mature biofilm with and without exposure to LCFS was determined by spot assay and enumeration of CFU, as depicted in the Log 10 reduction graph. (**B**) The quantification of total carbohydrate content from the biofilm with and without LCFS treatment. The bar graphs depict the measured OD values at 490 nm.

Biofilms are well known to provide a growth environment for cells within them, leading to the formation of persister cells that are resistant to antimicrobial agents. Viable cells from the preformed biofilm with and without LCFS treatment were enumerated using a spot assay on BHA plates. Biofilms without treatment (control) exhibited more than 8 ± 0.19 Log CFU/mL cells for mono- and dual-species biofilm in BHIB and more than 7.5 ± 0.21 Log CFU/mL in ASF conditions. In contrast, E-50 treatment reduced viability to up to 2 ± 0.16 Log CFU/mL sfor individual species and 3 ± 0.13 Log CFU/mL for dual species across all conditions. Similarly, when treated with LCFS, the number of viable cells in BHIB was below 4.5 ± 0.23 Log CFU/mL in mono- and 3.8 ± 0.16 Log CFU/mL in dual species. Whereas under ASF conditions the LCFS treated group exhibited about 3 ± 0.10 Log CFU/mL for *S. anginosus*, 3.3 ± 0.18 Log CFU/mL for *S. mutans* and around 3.6 ± 0.22 Log CFU/mL for dual species conditions (*p* < 0.05) (Fig. 5A). These results validate that LCFS has reduced the total viable cell count by more than 50% compared with the control.

### Effect of LCFS on EPS Inhibition

EPS from biofilm of both the species *S. mutans* and *S. anginosus* in individual as well as dual species state was extracted by the phenol: sulfuric acid method and quantified at 490 nm. Compared with the control, LCFS-treated biofilms in BHIB and ASF showed a decrease in total carbohydrate content in mono- and dual-species biofilms, as shown in Fig. 5B. This result clearly indicates that LCFS promotes EPS degradation, which might reduce biofilm formation and facilitate the disruption of mature biofilms.

## Discussion

Dental caries stands out as the most prevalent disease and remains a global burden. Additionally, dental caries is often associated with several systemic diseases, including diabetes, cardiovascular disease, infective endocarditis, and sepsis [38]. Mostly, *Streptococcal* species initiate biofilm formation on tooth surfaces, thereby providing a niche for other opportunistic pathogens, leading to dental caries [39]. Once, *Streptococcal* spp. like, *S. mutans* and *S. anginosus* form a biofilm matrix on tooth surfaces, become resistant to fluoride products currently used for treatment and as preventive measures to halt caries progression [21]. While these anti-caries agents are tolerated and have reduced impact on oral pathogens, different approaches are needed to overcome this crisis. For the past few decades, probiotics and postbiotics have attracted significant attention among researchers for their numerous applications supporting host health [40]. These beneficial microbes and their metabolites are arising as valuable alternatives as they are well reported to combat gastrointestinal [41], respiratory [10, 42, 43], and urogenital infections caused by pathogenic microorganisms [44]. Many probiotics like *Lactobacillus* (*L. rhamnosus* GG, *L. casei* Shirota, *L. plantarum* 299v, and *L. acidophilus* DDS-1) and *Bifidobacterium* strains (*B. lactis* BB-12, *B. lactis* CP-9, *B. longum* BB536, *B. longum* BORI, and *B. breve* MCC1274) are well studied, commercialized, and certified as Generally Regarded as Safe (GRAS) by the FDA. Similarly, many other probiotic strains, such as *Streptococcus thermophilus*, *Bacillus coagulans*, *Bacillus subtilis*, *Pediococcus acidilactici*, and *Pediococcus pentosaceus*, have been reported to possess several beneficial properties that favor human health [45, 46]. On the other hand, the use of live probiotics affects immunocompromised individuals; postbiotics do not and are recognized as safe. They are not live microorganisms, but metabolites produced by beneficial microbes [47]. In particular, *Pediococcus* strains and their metabolites have been extensively reviewed for numerous biological activities, including antibacterial, antifungal, anti-inflammatory, and antioxidant properties, yet they have not been used satisfactorily [48, 49]. In this regard, *Pediococcus pentosaceus* GS4, isolated by our research group, has been studied for more than a decade for its biological properties and has shown antioxidant, anti-inflammatory, antimicrobial, and anticancer activities [50]. Several reviewers have evaluated its potential for use in various beneficial applications [48]. Considering the potential of our probiotic strain, *P. pentosaceus* GS4, and the need for new therapeutic approaches to prevent and treat dental caries, we investigated the antibacterial and antibiofilm properties of the postbiotics component, lyophilized cell-free supernatant, of *P. pentosaceus* GS4 against oral pathogens. Cell-free supernatant consists of metabolites produced by the probiotic strain, like organic acids, antimicrobial peptides, bacteriocin, extracellular enzymes, surface-layer proteins, and exopolysaccharides. Also, the postbiotic preparations are easy to prepare and technologically viable. Here, the CFS was lyophilized to obtain a more concentrated metabolite. Treated LCFS showed reduced anti-cariogenic properties due to the denaturation of some metabolites. First, the LCFS was evaluated for antibacterial activity against *S. mutans* and *S. anginosus*. The results showed that LCFS at 400 mg/mL completely inhibited the growth of both species, as determined by kill curve analysis (Fig. 1B-ii). This demonstrates the potential antibacterial effect of LCFS against these oral pathogens within 24 h, making it a suitable preventive agent in the treatment of dental caries and other oral infections. Similar research studies have been carried out using the LCFS of *Lactobacillus* strains against *E. faecalis* and found 300 mg/mL to be effective against the pathogen [51, 52]. Also, erythromycin was used in this study instead of clinical agents such as fluoride and chlorhexidine to validate the *in vitro* assays. Similarly, erythromycin acts as a higher-efficacy inhibitor of pathogens, serving as a baseline for quantifying the inhibitory capacity of the metabolites present in the LCFS, ensuring direct comparison between the pharmaceutical-grade agent and the experimental compound.

The initial step for a bacterium to colonize the oral cavity is to adhere to tooth surfaces and initiate biofilm formation, which leads to the development of caries [53]. Adherence begins with reversible, weak binding to the enamel pellicle and then progresses to irreversible adhesion. This strong adhesion leads to subsequent steps, including biofilm formation and maturation, EPS production, and acid production, resulting in demineralization of tooth enamel [54]. In this study, LCFS was assessed for its effect on the adherence of *S. anginosus* and *S. mutans* to glass tubes resembling the tooth surface. There was a significant reduction in adherence of approximately 70% in *S. anginosus* across all three conditions, and above 65% in *S. mutans* (Fig. 4A). Along with adherence, hydrophobicity is another significant factor in host-pathogen interactions [55]. Several studies have suggested that reducing the hydrophobicity of these *Streptococcal* spp. obstructs their adherence to hard surfaces [56]. A study by Kim *et al*. (2022) reported that the CFS of *L. brevis* KCCM 202399 exhibited 40% inhibition of *S. mutans* cell surface hydrophobicity by the MATH (Microbial Adhesion to Hydrocarbons) test [21]. Likewise, the CFS of *L. plantarum* Ln4 was evaluated for its role in altering the cell-surface hydrophobicity of *S. mutans*, and a 16% reduction was observed [57]. In the present study, we found that LCFS decreased hydrophobicity and exhibited a higher inhibition rate than the control (Fig. 4B). These two results suggest that LCFS can reduce the adherence capacity and lower the hydrophobicity of caries-causing pathogens by interfering with the initial attachment of oral pathogens to teeth and with plaque formation.

Biofilms are composed of various components, including extracellular polysaccharides, proteins, lipids, and eDNA. These components help bacteria form a thick, film-like matrix and provide niches for cells to reside and tolerate harsh conditions [58]. Streptococcal biofilms prevent penetration and resist the entry of antimicrobial agents used for treatment and prevention [18]. Recent research emphasises the need for both anti-bactericidal and antibiofilm agents against these pathogenic dental plaques. Several studies have been carried out mainly to inhibit these pathogenic biofilms of *S. mutans* by employing herbal extracts [59-61], essential oils [62-64], metal ions [65, 66], antibiotics [67], bacteriophage [68], and a few studies with probiotics [69-71]. Also, postbiotics have been widely studied for their antibacterial and antibiofilm potential against pathogens that cause various infections. Several studies have shown that postbiotics inhibit biofilms formed by oral pathogens, particularly *S. mutans*, under *in vitro* conditions. Mainly, the cell-free supernatant of *L. plantarum* ATCC 11741 was effective in inhibiting *S. mutans* biofilm formation with 85% inhibition, and the CFS of *L. salivarius* MG4265 exhibited 73% inhibition [72, 73]. Similarly, Ciandrini *et al*. (2016) reported that biosurfactants in the CFS from *L. acidophilus* DDS-1 exhibited 98% of inhibition and 99% for *L. reuteri* DSM 17938 [74]. Recently, a study by [75] Niranjan *et al*. (2024) tested the anti-biofilm potential of *L. rhamnosus* ATCC 7469 against *S. mutans* and observed around 80% inhibition. Although these and several other studies have reported the anti-biofilm effect only under in vitro conditions, the present study examined it under both *in vitro* and simulated conditions. To mimic the physiological environment of the oral cavity, we evaluated the LCFS's role in biofilm inhibition in simulated salivary fluid at pH 5.5 and 6.8, which demonstrated above 65% biofilm inhibition for *S. anginosus*, 63% for *S. mutans*, and more than 60% in dual-species conditions, indicating effective antibiofilm activity.

Biofilm inhibition is a key factor for an anti-biofilm agent, but disruption of a mature biofilm is another indicator of a compound in treatment strategies. Wasfi *et al*. (2018) reported that CFS of *L. salivarius*, *L. plantarum*, and *L. reuteri* exhibited 47%, 26%, and 24% eradication of preformed biofilm, respectively [76] . In a study, CFS of *L. plantarum* 108 caused 36% biofilm disruption in *S. mutans* and 50% in mixed-species (*S. mutans* and *Candida albicans*) biofilms [68]. Jung *et al*. (2021) evaluated the role of CFS from eight different *Lactobacillus* spp. against *S. mutans* and its biofilm. CFS of *L. lactis* MG5125, *L. salivarius* MG4265, *L. casei* MG311, and *L. rhamnosus* MG316 possessed above 75% of preformed biofilm eradication [73]. Similarly, Kim *et al*. (2022) studied the antibiofilm effects of cell-free supernatants from *Lactobacillus brevis* strains on *S. mutans* biofilms and found around 50% biofilm disruption [21]. LCFS, in this study, demonstrated biofilm disruption above 65% in mono-species and 61% in dual-species conditions in BHIB, and 75% in both mono- and dual-species conditions in ASF. These results were confirmed with different microscopic analyses. SEM analysis revealed no structural damage to the cells, only a reduction in population (Fig. 3B). Similarly, in LIVE/DEAD staining, the dead cells were found to be in higher amounts, indicating the ability to inhibit the growth of oral pathogens in a dual state. Additionally, LCFS-treated biofilm had fewer viable cells than the control, indicating complete eradication of the biofilm and persister cells (Fig. 3A).

*Streptococcus* spp. possesses physiological traits such as EPS synthesis, adaptation to low pH conditions, and resistance to oxidative stress. These traits primarily contribute to demineralization and to caries progression when these species become resistant to low pH and colonize onto tooth surfaces [75]. Also, these species tolerate acidic conditions within their multi-layered biofilm, using EPS as an important physical barrier, thereby subjecting bacterial cells to low pH [76]. Considering this, a study by Kim *et al*. (2022) reported that CFS of *L. brevis* KCCM 202399 inhibited EPS production by 67%, thereby affecting the structural integrity of the biofilm [21]. Similarly, Jang *et al*. (2024) reported that *L. plantarum* Ln4 exhibited approximately 35% inhibition of total EPS [57]. In our study, we quantified EPS from biofilms (control and LCFS-treated) and found that LCFS significantly reduced EPS production by around 60% across all three conditions (Fig. 5B). This indicates that LCFS can reduce EPS production by *Streptococcus* species, thereby mitigating enamel degradation. In addition to biofilm formation and EPS production, the aciduric properties of *S. mutans* are among the most critical pathogenic factors. *Streptococcal* spp. metabolize dietary sugars and thrive in low-pH environments, altering dental biofilms and favoring acid-tolerant species, which results in enamel demineralization. Our findings indicate that, in the absence of LCFS exposure, *Streptococcus* spp. survive at pH 5 for longer and adapt to the acidic condition, whereas in the LCFS-exposed cells, the number of viable cells was reduced. Additionally, *S. mutans* can remain active under oxidative stress, which contributes to its virulence in causing caries [79]. Here, both test pathogens were resistant to lower H_2_O_2_ levels in the control. Still, after treatment with LCFS, both *S. mutans* and *S. anginosus* were sensitive to lower H_2_O_2_ levels, indicating that LCFS sensitized the species to H_2_O_2_ (Fig. 4C). As LCFS was found to reduce the EPS synthesis, this increase in sensitivity towards H_2_O_2_ can be due to destabilization of the EPS matrix, leading to easy penetration of H_2_O_2_ into the membrane of cells. A similar study carried out by Wen *et al*. (2017) reported that *L. casei* grown along with *S. mutans* displayed higher sensitivity to H_2_O_2_, whereas in the absence of *L. casei*, the *S. mutans* was found to be resistant to H_2_O_2_ [78]. These findings emphasize that LCFS inhibited virulence traits in *Streptococcus* species, as demonstrated by a series of microbiological assays. Gene-level regulatory studies are needed to elucidate the underlying mechanisms of antibiofilm effects and anticariogenic properties in the near future.

## Conclusion

Probiotics and postbiotic effects on oral health are being studied widely. Among pathogenic bacteria, *S. mutans* plays a prominent role in dental caries, and *S. anginosus* is often associated with it. *P. pentosaceus* GS4, a well-studied probiotic strain, demonstrated a wide range of effects and other applications. In this study, the lyophilized cell free supernatant of the strain were assessed for their effects on these two pathogens. Altogether, the results from this study suggest that LCFS from the probiotic *P. pentosaceus* GS4 exhibits antibacterial, antibiofilm, and antivirulence effects against *S. mutans* and *S. anginosus* under artificial salivary conditions that mimic the oral environment, making it a promising compound for inhibiting these pathogens. Further, these findings require molecular and *in vivo* studies to validate the current findings, and can aid in the future for incorporating this probiotic preparation into daily-use products such as mouthwash and toothpaste, which could be a strategy for preventing and treating caries.

## Figures and Tables

**Table 1:** List of components that are used in the preparation of artificial saliva employed in this current study.

S. No	Components	Quantity (in g/L)
1	Potassium chloride (KCl)	0.96
2	Sodium chloride (NaCl)	0.674
3	Magnesium chloride (MgCl_2_·6H_2_O)	0.0405
4	Calcium chloride (CaCl_2_·2H_2_O)	0.117
5	Potassium dihydrogen phosphate (K_2_HPO_4_)	0.091
6	Methyl parahydroxybenzoate (C_8_H_8_O_3_)	0.11
7	Sorbitol (C_6_H_14_O_6_)	8.0

**Table 2:** **Antibacterial activity of LCFS against *S. anginosus* and *S. mutans* at varying concentrations (100 to 500 mg/mL), erythromycin (50 μg/mL) is used as a positive control, and 1× PBS as the negative control.** ZOI is measured and expressed as the mean ± Std (mm). '-' represents no activity. Std-standard deviation

Concentration of LCFS (mg/mL)	Zone of inhibition (in mm)	
	*S. anginosus*	*S. mutans*
Erythromycin (50 μg/mL)	30 ± 0.36	23 ± 0.20
1× PBS	-	-
100	-	-
200	-	-
300	11 ± 0.43	-
400	15 ± 0.20	16 ± 0.25
500	21 ± 0.20	20 ± 0.58

**Table 3 T3:** Effects of pH, temperature, and proteinase-K treatment of LCFS.

Parameters		Zone of inhibition (ZOI in mm)		Metabolic activity (%)	
		*S. anginosus*	*S. mutans*	*S. anginosus*	*S. mutans*
Treated	pH neutralized	11 ± 0.14	10 ± 0.14	64	79
	Proteinase K	12 ± 0.14	12 ± 0.28	25	34
	Heated at 60°C	10 ± 0.21	11 ± 0.14	21	32
	80°C	10 ± 0.07	10 ± 0.14	28	34
	100°C	10 ± 0.141	12 ± 0.21	77	81
	121°C	-	-	92	97
Untreated	LCFS	15 ± 0.32	16 ± 0.20	8	6
